# Urgent and emergent pediatric cardiovascular imaging

**DOI:** 10.1007/s00247-024-05980-y

**Published:** 2024-07-05

**Authors:** Charlotte de Lange, Carlos Marin Rodriguez, Claudia Martinez-Rios, Christopher Z. Lam

**Affiliations:** 1 Department of Pediatric Radiology, Queen Silvia Children’s Hospital, Sahlgrenska University Hospital, Behandlingsvägen 7, 416 50, Gothenburg, Sweden; 2 Institute of Clinical Sciences, Sahlgrenska Academy, University of Gothenburg, Gothenburg, Sweden; 3 Radiología Pediátrica, Hospital Universitario Gregorio Marañón, Madrid, Spain; 4 Department of Diagnostic and Interventional Radiology, The Hospital for Sick Children, Toronto, ON Canada; 5 Department of Medical Imaging, University of Toronto, Toronto, ON Canada

**Keywords:** Cardiovascular system, Computed tomography, Congenital heart defect, Emergencies, Imaging, Pediatric

## Abstract

The need for urgent or emergent cardiovascular imaging in children is rare when compared to adults. Patients may present from the neonatal period up to adolescence, and may require imaging for both traumatic and non-traumatic causes. In children, coronary pathology is rarely the cause of an emergency unlike in adults where it is the main cause. Radiology, including chest radiography and computed tomography in conjunction with echocardiography, often plays the most important role in the acute management of these patients. Magnetic resonance imaging can occasionally be useful and may be suitable in more subacute cases. Radiologists’ knowledge of how to manage and interpret these acute conditions including knowing which imaging technique to use is fundamental to appropriate care. In this review, we will concentrate on the most common cardiovascular emergencies in the thoracic region, including thoracic traumatic and non-traumatic emergencies and pulmonary vascular emergencies, as well as acute clinical disorders as a consequence of primary and postoperative congenital heart disease. This review will cover situations where cardiovascular imaging may be acutely needed, and not strictly emergencies only. Imaging recommendations will be discussed according to the different clinical presentations and underlying pathology.

**Graphical Abstract:**

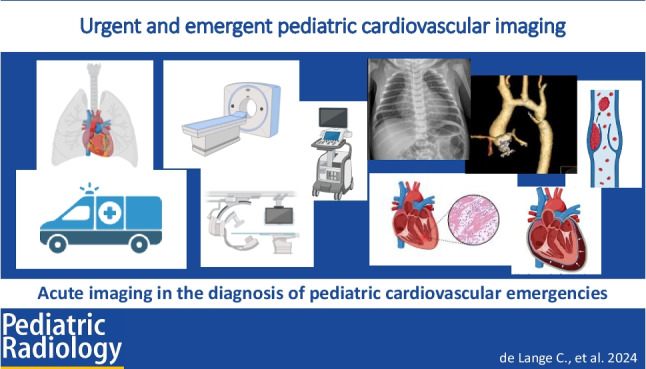

## Introduction

Cardiovascular emergencies are rare in children, although common in the adult setting. While in adults, the most common symptom is chest pain in coronary artery disease, in children, the clinical presentation ranges from mild symptoms with no consequences to life-threatening events. Emergencies can be divided into acute events in patients with known or unknown congenital heart disease and acquired diseases. The diagnosis and management of these patients rely on skilled emergency clinicians, as well as pediatric intensive care and cardiology specialists. Diagnostic imaging may be required, with chest radiography and echocardiography being the first step, and computed tomography (CT) and cardiovascular magnetic resonance imaging (CMRI) occasionally necessary. This review will cover the imaging approach to the more common selected cardiovascular emergencies and acute conditions in children, including imaging findings of acute complications in patients with congenital heart disease, anomalies of pulmonary vessels, and acute traumatic and non-traumatic acquired cardiovascular diseases in children.

## General imaging approach

Choice of imaging will depend on the nature of the cardiovascular emergency, age and size of the patient, and local institutional practice and resources. Radiographs are typically first line and may be the initial clue to an emergent finding. However, cardiovascular emergencies often will require further imaging assessment. Echocardiography is widely accessible and effective in detecting many intracardiac and pericardial pathologies, including inlet/outlet obstructions, ventricular dysfunction, or pericardial tamponade. Cardiovascular CT is highly suitable for emergent imaging as it provides rapid evaluation of cardiovascular anatomy and can usually be successful without the need for general anesthesia if using a modern scanner [1–3] and thus will often be the next-step imaging modality of choice. Cardiac MRI is largely reserved for trouble shooting or refining diagnoses in complex cases. Table 1 summarizes the strengths and weaknesses of, and indications for the different modalities.

**Table 1:** Imaging modalities used in cardiovascular emergencies

Modality	Advantages	Disadvantages	Indications in the acute setting
Radiography	• Widely available • Fast	• Limited intracardiac and cardiovascular information	• Screening for alternative diagnoses (e.g., lung disease) • Assessment of pulmonary vascularity
Echocardiography	• Widely accessible • No contrast media required • Highest temporal resolution • Ventricular and valvar function	• Limited views in some patients • Suboptimal assessment of extra-cardiac structures • Poor tissue characterization	• Cardiomyopathy/myocarditis • Complications of CHD • Pericardial disease
CT	• Fast • Excellent spatial resolution • Global anatomic information	• Ionizing radiation • Lowest temporal resolution	• Trauma • Stenoses/obstructions in CHD • Postoperative complications in CHD • Coronary assessment • Embolic disease
MRI	• Tissue characterization • Scar imaging • Gold standard for ventricular function and flow hemodynamics	• Limited access and expertise • Resource intensive • Longer exam time and greater need for sedation in young children	• Cardiac mass • Vasculitis • Cardiomyopathy/myocarditis • Pericardial disease
Cardiac Catheterization	• High spatial resolution • Pressure measurements • Ability to perform concomitant intervention	• Invasive • Ionizing radiation • Need for sedation	• Assessment and intervention for cardiovascular obstruction (coronary, ductal, valvular, pulmonary, aortic)

*CHD* congenital heart disease, *CT* computed tomography, *MRI* magnetic resonance imaging

### Cardiovascular computed tomography technique considerations

Cardiovascular CT technique will depend on the CT system available to each institution. The primary goal is to obtain complete coverage with adequate concentration of intravascular contrast media in the region of interest, using as fast a temporal resolution and acquisition speed as possible, which may or may not require cardiac gating. Most modern scanners can reliably diagnose extra-cardiac cardiovascular pathology even without gating [4–6] so if coverage is an issue, for example, with volumetric scanners in the setting of multisystem trauma, it is reasonable to perform a helical acquisition without gating. Repeat dedicated cardiac-gated CT is rarely necessary in either situation. High pitch scanning via dual-source scanners is particularly robust to motion and pulsation artifacts, which can be performed with or without electrocardiography (ECG)—gating and without coverage limitations [4]. However, cardiac gating is preferred for coronary or intracardiac assessment in the pediatric population given the faster heart rates in children [7] and can also be useful for assessment of the aortic root and main pulmonary trunk with older generation scanners.

Contrast injection strategy varies based on the indication. Modern CT scanners are often capable of diagnostic quality cardiovascular imaging even without completely maximizing intravascular contrast media, such as with a conventional computed tomography angiography (CTA) technique. This is possible because of the improved acquisition speed combined with the ability to perform low kV and high mA scanning with modern scanners, which reduces motion artifacts and increases image contrast, respectively. Depending on the indication then, it may be worthwhile to prolong the injection time or image after a slight delay to homogenously opacify both the pulmonary and systemic circulations, or to better assess associated soft tissue and parenchymal enhancement. CT imaging in the setting of a bidirectional cavopulmonary connection or Fontan circulation requires a larger volume of contrast (3–4 cc/kg) with an extended delay. In Fontan circulation, injection of contrast media can be via the foot with delay of at least 30–60 s to opacify the bidirectional cavopulmonary connection via recirculation from the head and neck. Upper extremity injection in bidirectional cavopulmonary connection is also possible but may be contaminated by undesirable streak artifacts or heterogenous opacification if not timed precisely, and contrast media will be diluted by both the pulmonary and systemic capillary beds in upper extremity recirculation imaging (unlike with a lower extremity injection which bypasses the pulmonary circulation). In Fontan circulation, the delay should be at least 60–90 s [8]. In patients with extracorporeal membrane oxygenation (ECMO), many different injection strategies can be successful. Simply injecting a higher volume of contrast in a peripheral vein is the simplest method and is usually sufficient. If the patient can tolerate slowing the ECMO circuit for a brief period, this can also be helpful. Finally, it is also possible to power inject into the venous cannula of the ECMO circuit. In general, for all contrast injection strategies, the exact timing of imaging can be variable depending on patient size, cardiac output, anatomy, and injection rate chosen.

### Cardiovascular magnetic resonance imaging technique considerations

Access to urgent or emergent CMRI will depend on institutional resources and expertise. These scans should only be performed in urgent or emergent situations in patients who can safely tolerate a scan and when the results are likely to change immediate management. A multidisciplinary team should be aware of the case with support staff available if needed. It may be necessary to tailor the protocol to be as short as possible as often only one or two specific pieces of information are needed. Access to highly accelerated, motion compensated, or multiparametric sequences may be particularly useful in such urgent scenarios.

## Imaging of acute complications in patients with congenital heart disease

While any child can present with a cardiac emergency, patients with a structural anomaly of the heart and vessels are at increased risk for developing acute life-threatening events. Furthermore, the refinements in surgical techniques and peri-/postoperative care have resulted in increased survival of children with more severe and complex congenital heart disease. Such patients may present with various complex acute conditions or complications [9].

### Neonatal period

Most congenital heart diseases, and especially critical anomalies, are nowadays identified prenatally by fetal cardiac ultrasound in about 75–80% of cases [10, 11]. However, some will still present undiagnosed at the emergency department after discharge from hospital [12]. Infants with severe congenital heart disease can be asymptomatic with a normal physical examination at discharge due to patency of the ductus arteriosus. The duct usually closes within 1 to 2 days of life but may be persist for about 1 week [13]. In congenital heart disease dependent on ductal flow, ductal closure can cause a critical hemodynamic change. Any child ≤ 6 weeks of age presenting with symptoms of shock and cyanosis should raise suspicion for closure of the duct in a right- or left-sided obstructive cardiac anomaly [14, 15].

The left-sided obstructive lesions comprise of hypoplastic left heart syndrome, critical aortic stenosis, interrupted aortic arch, and coarctation of the aorta, overall representing about 12.4% of congenital heart disease [14]. Coarctation of the aorta is an especially difficult diagnosis to make by prenatal ultrasound and might be missed [16], as the coarctation may not become manifest until the duct fully closes. Babies with coarctation may have symptoms of pulmonary over circulation (due to atrial, ventricular, or ductal shunting), systemic hypoperfusion, circulatory collapse, and shock. Similar symptoms may present in patients with obstructed anomalous pulmonary venous connections [17]. Milder coarctations of the aorta present later in life and rarely as emergencies, more often discovered as a secondary finding or during the work-up of hypertension.

Right-sided obstructive lesions dependent on ductal patency comprises mainly of severe tetralogy of Fallot with pulmonary atresia and ductal pulmonary origin, pulmonary atresia with intact ventricular septum, other forms of complex congenital heart disease (atrioventricular septal defects, double outlet right ventricle, double inlet left ventricle, etc.) with pulmonary atresia, or some forms of tricuspid atresia. Prostaglandin infusion is mandatory to ensure pulmonary blood flow via the ductus arteriosus to the pulmonary artery and in specific cases, surgical shunting or ductal stenting can be performed as palliative procedures [18]. In transposition of the great arteries, the pulmonary and systemic circulation are in parallel instead of serial, with the aorta arising from right ventricle and the pulmonary artery from the left. Adequate intracardiac mixing ± ductal shunting is obligate for survival and may require balloon atrial septostomy to establish or ensure an adequate atrial level shunt until the repair operation, the arterial switch operation, can be performed, where the pulmonary artery and the aorta are “switched” back, and the coronary arteries are reimplanted.

**Fig. 1:**
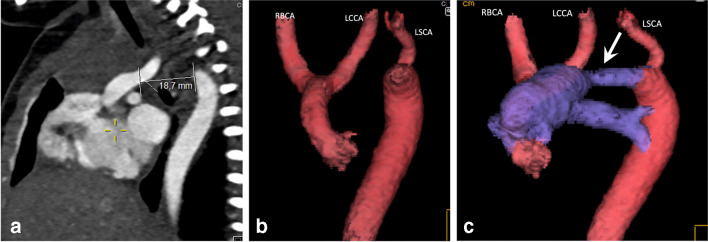
Contrast-enhanced computed tomography angiography images in a newborn boy with circulatory collapse on day one of life, in whom echocardiography showed poor quality views of the aortic arch. **a** Sagittal contrast-enhanced image shows a long gap (18.7 mm) between the interrupted aortic arch and the descending aorta. **b**, **c** Anteroposterior (**b**) and sagittal oblique (**c**) volume rendered images with the ascending and descending aorta and branches in red and pulmonary artery, left pulmonary artery, and open arterial duct (*arrow in* **c**) in blue. The open arterial duct secures blood flow to the descending aorta. The left subclavian artery (LSCA) arises from the descending aorta next to the arterial duct. All images (**a–c**) show a type B interrupted aortic arch occurring just after the right brachiocephalic artery (RBCA) and the left common carotid artery (LCCA)

**Fig. 2:**
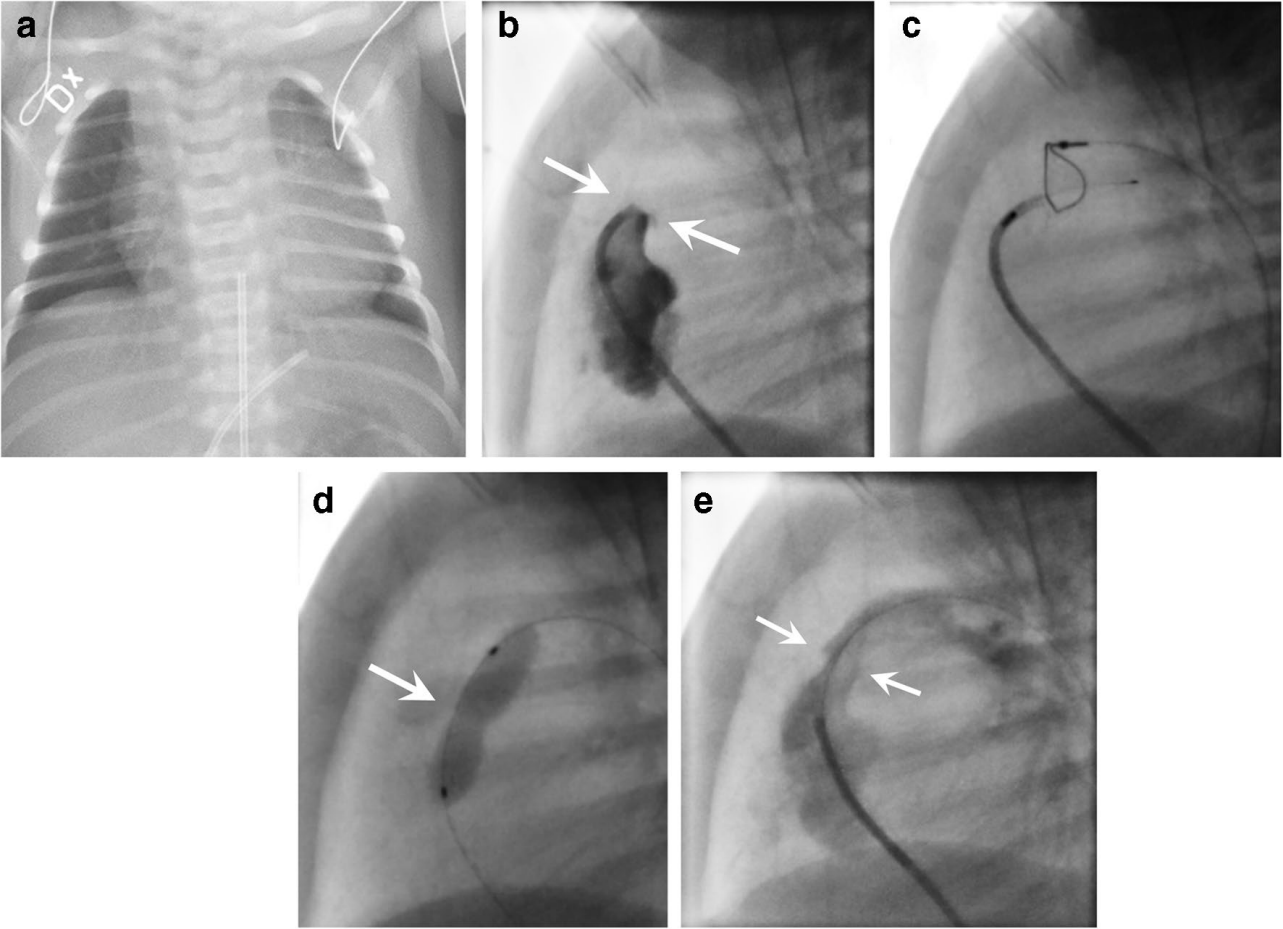
A newborn boy with a prenatal diagnosis of pulmonary atresia with intact ventricular septum. The diagnosis was confirmed by echocardiography after birth and prostaglandin infusion was started immediately to assure patency of the arterial duct. **a** Anteroposterior chest radiograph shows an enlarged heart and small size pulmonary vessels. Two umbilical catheters (one arterial and one venous are positioned in the midline subdiaphragmatically). **b** Lateral view from cardiac catheterization performed on day 3 of life shows the atretic pulmonary valve (*arrows*). **c**, **d** Treatment with radiofrequency perforation and a snare to open the valve (**c**) followed by balloon dilatation of the valve (*arrow in* **d**). **e** Lateral angiograph view after dilatation, reveals a successful opening of the pulmonary valve (*arrows*) and the pulmonary artery. Case courtesy of Dr Janus Freyr Gudnason, Queen Silvia Children’s Hospital, Gothenburg, Sweden

In the above cases, echocardiography will always be the first-line examination together with chest radiography before medical, surgical, or interventional treatment is started. Additional anomalies of the coronaries and the aortic arch, coarctation, or pulmonary arteries and veins might require further acute preoperative planning with CT (Fig. 1) [19]. Sometimes cardiac catheterization is performed for interventional treatment with dilatation of critical aortic or pulmonary stenosis and ductal stenting, and may then provide the necessary anatomical and hemodynamic data [18] (Fig. 2).

### Beyond the neonatal period

**Fig. 3:**
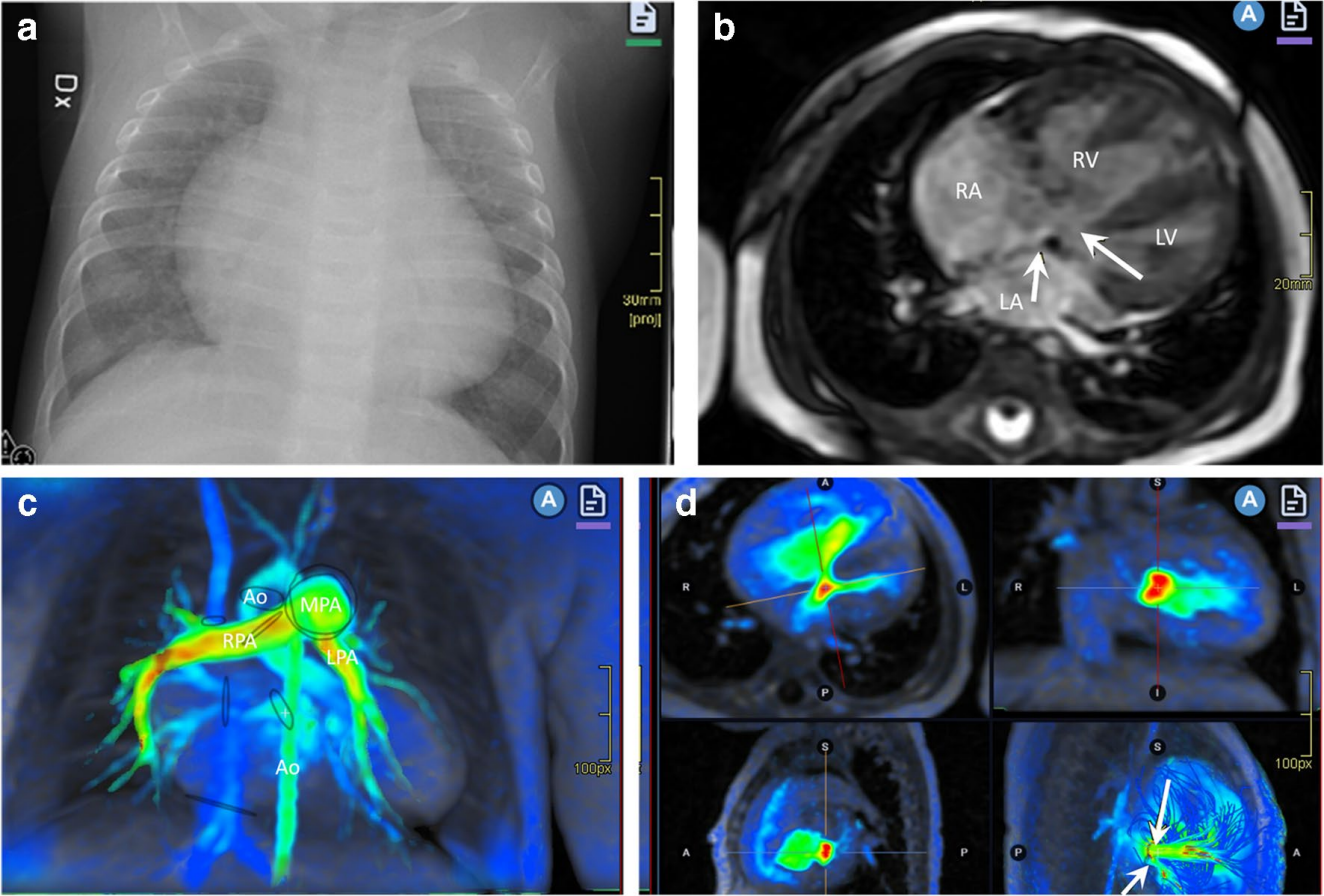
A 7-month-old girl presenting with acute cardiac failure due to a previous undiagnosed incomplete atrioventricular septal defect with mitral stenosis and a small left ventricle. **a** Anteroposterior chest radiograph shows an enlarged heart with congestive pulmonary vessels. **b** Magnetic resonance 2-dimensional (D) steady state free precession image in a 4-chamber view reveals an enlarged right ventricle and right atrium, the atrial primum defect (*short arrow*) and ventricular septal defect (*long arrow*). **c**, **d** A 4-D phase contrast flow sequence (4-D flow) where the color coding represents blood flow velocity; from low velocity in blue to high velocity in red. **c** A volume rendered image with traced regions of interest on the different vessels, valves, and shunts showing an enlarged main pulmonary artery and branches. **d** Multiplanar reconstruction with regions of interest marked on the septal defects (*arrows*). The 4-D flow sequence enabled visualization of the mitral stenosis (*red color*) and septal shunts as well as and direct quantification of the shunts with Qp:Qs = 4:1. *Ao* aorta, *LA* left atrium, *LPA* left pulmonary artery, *LV* left ventricle, *MPA* main pulmonary artery, *RA* right atrium, *RPA* right pulmonary artery, *RV* right ventricle

Other congenital heart diseases not dependent on ductal flow will appear later around 4–6 weeks after birth as the pulmonary vascular resistance has fallen. Defects in the atrial or ventricular septum (ASD, VSD) can then become symptomatic and acute congestive heart failure will develop as a result of a significant left to right shunt and pulmonary congestion. Infants typically present with poor feeding, tachypnea, sweating, and failure to thrive. If undiagnosed, the pulmonary vascular resistance increases, and the shunt may reverse to right to left shunting, causing cyanosis with multisystem affection, known as Eisenmenger syndrome. These septal defects are evaluated with echocardiography in the acute/subacute setting, while CTA or CMRI can provide additional information if there is suspicion for anomalous pulmonary veins (e.g., if not well seen or in sinus venosus ASDs) or scimitar syndrome [17]. MRI using 2-dimensional (D) or 4-D flow quantification can be used in the subacute workup for preoperative evaluation of the shunt(s) and ventricular volumes [20, 21] (Fig. 3).

### Postoperative cardiovascular complications in congenital heart disease

**Fig. 4:**
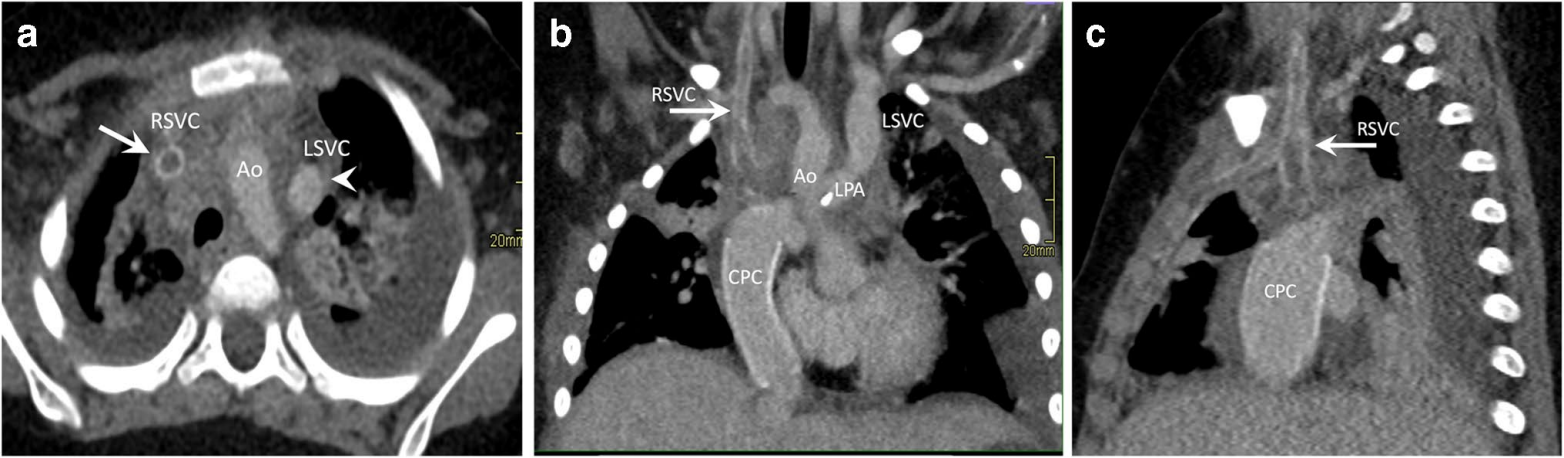
A 2-year-old girl, presenting with acute symptoms with low oxygen saturation 3 weeks after surgical repair to a total cavopulmonary connection. A right and left superior vena cava were anastomosed to the respective pulmonary arteries (bilateral Glenn procedure). Echocardiography did not reveal flow in the right superior vena cava. **a–c** Contrast-enhanced computed tomography with multiplanar reconstructions in axial (**a**) coronal (**b**) and sagittal (**c**) planes shows a patent left superior vena cava (SVC) with high density contrast in the lumen connected to the left pulmonary artery (*arrowhead* in **a**) and confirms the presence of an occluding thrombosis with low density and rim enhancement in the right SVC (*arrow in* **a–c**). *Ao* aorta, *CPC* cavopulmonary connection to the pulmonary arteries with a conduit from inferior vena cava, *LSVC* left superior vena cava, *LPA* left pulmonary artery, *RSVC* right superior vena cava

**Fig. 5:**
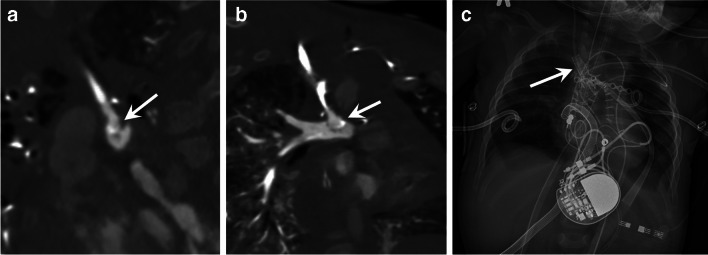
A 9-month-old boy with double outlet right ventricle, surgically repaired with a partial cavopulmonary shunt (Glenn procedure) and extracorporeal membrane oxygenation (ECMO) treatment. The patient was hemodynamically unstable. Contrast-enhanced pulmonary computed tomography angiography with multiplanar reconstructions reformatted images in the sagittal (**a**) and coronal (**b**) planes show a linear endoluminal lesion consistent with pulmonary artery dissection (*arrows*). **c** Postoperative anteroposterior chest radiograph showing endotracheal tube, ventricular assist cannulae, a pacemaker and pleural drainage catheters, and a stent placed at the Glenn anastomosis (*arrow*)

In congenital heart disease, postoperative complications may present early or late with thrombotic events in vessels, bleeding, aneurysms, dissections, and patch detachments as well as infections in relation to grafts or implants (pulmonary graft and coarctation). These may present with acute symptoms requiring immediate CT angiography if the patient is stable [22]. Emergencies in patients with a *functional single ventricle* require specific attention due to the unique physiology. The surgical repair of these patients is performed in three steps from the neonatal period to the final Fontan circulation at 2–4 years of age, where the systemic venous return is directed passively via the caval veins to the pulmonary arteries [23]. Acute complications may occur in the early postoperative periods or later on. A feared complication in these patients is an occlusive thrombus in the bidirectional cavopulmonary connection, the Fontan tunnel, or in the anastomosis of the native aorta to the neoaorta, i.e. the former pulmonary artery in hypoplastic left heart syndrome, which can result in severe hemodynamic changes. These complications are often best demonstrated by CT with attention to contrast injection and timing (Figs. 4 and 5) [24].

## Acute anomalies of the pulmonary vessels

Acute anomalies of the pulmonary vessels encompass a wide range of acquired and congenital diseases affecting the pulmonary veins and arteries. While pulmonary embolism is a common emergency in adults, it is relatively uncommon in children. Conversely, obstruction of the pulmonary arteries or veins in neonates with congenital heart disease is not uncommon. Extrinsic masses or vessel wall diseases (e.g., vasculitis, neoplasms), pulmonary artery aneurysms, and iatrogenic lesions can cause acquired occlusions of the pulmonary arteries, which may present as a cardiovascular emergency.

Echocardiography often has limitations in evaluating pulmonary vessels due to suboptimal acoustic windows and pulmonary air. Therefore, computed tomography is generally the preferred imaging method for emergencies of the pulmonary vessels. Although MRI has been shown to be accurate in diagnosing pulmonary artery conditions in both adults [25] and children [26, 27], it has drawbacks when compared to CT scans. These include prolonged examination times, limited availability in many centers, incompatibility with diverse life support devices, and lower accuracy for alternative diagnoses, particularly lung parenchymal diseases. However, in certain situations where a comprehensive examination is required, as in complex congenital heart diseases (e.g., heterotaxy syndromes), and patients are stable, MRI can be considered.

### Pulmonary embolism

Pulmonary embolism was previously considered to be an uncommon disease in the pediatric population. Albeit still infrequent, pulmonary embolism is now reported to be more common in autopsy series than previously thought [28]. However, diagnosis in clinical practice is still challenging, in part because of nonspecific clinical findings and inaccurate laboratory tests [29–31]. Diagnostic criteria for pulmonary embolism in adults, such as elevated heart rate, low blood oxygen saturation, or clinical signs and symptoms, are less specific in children. Diagnostic tests such as D-dimer levels [32] and ECG findings have not been validated in children. Therefore, a high index of suspicion is required to diagnose pulmonary embolism in children.

**Fig. 6:**
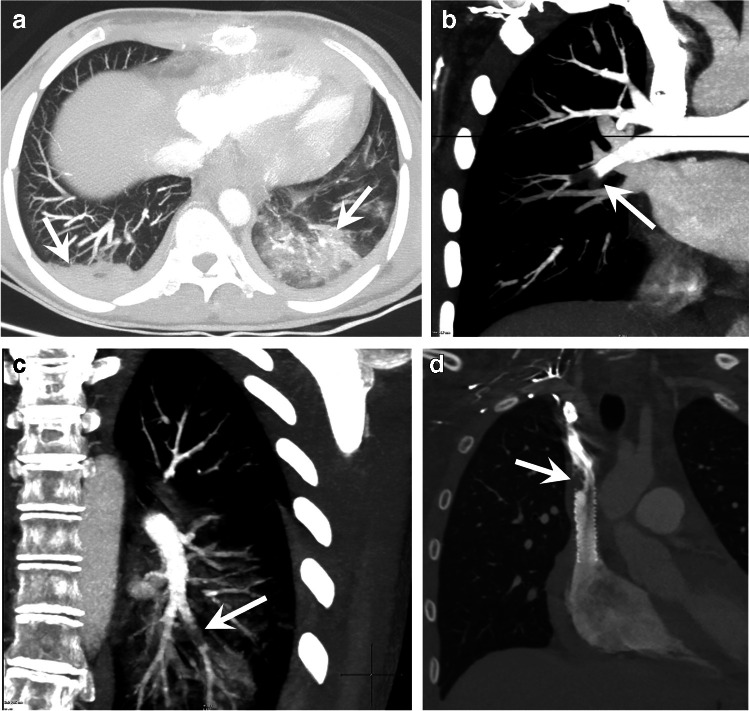
A 9-year-old girl with a heart transplant and a Dacron conduit in the superior vena cava (SVC). The patient presented with acute hypoxemia and pulmonary infiltrates. A contrast-enhanced computed tomography of the thorax was ordered to rule out pulmonary embolism. Bilateral pulmonary consolidations were seen on an axial maximum intensity projection (MIP) image (*arrows* in **a**). Angiographic coronal MIP images show occlusive thrombi in the pulmonary arteries of the lower lobes (*arrows*) in the right lung (**b**) and the left lung (**c**). A conduit-associated thrombus was seen on a higher window in coronal multiplanar reconstruction CT image (*arrow* in **d**)

There is a bimodal age peak in pediatric pulmonary embolism, being more common in neonates and adolescents. Central venous lines, complex medical conditions, congenital heart disease, immobilization, prothrombotic states, and oral contraceptives are known risk factors, whereas idiopathic pulmonary embolism in children is very rare. In most cases, a child with pulmonary embolism has a combination of two or more risk factors (e.g., oral contraceptives and obesity, cardiac patient with a central line, trauma patient with a prothrombotic state). There are certain clinical situations in which pulmonary embolism is highly suspected. For example, clinicians are more alert for embolism in patients with congenital heart disease with endovascular devices and hypoxemia (Fig. 6).

Patients with sickle cell disease, especially those with acute chest syndrome, are at a higher risk for pulmonary embolism. The causes of acute chest syndrome are varied and can include infection, bland pulmonary embolism, or fat emboli. Although there used to be concern about a possible association between iodinated contrast and sickling, it has been shown to be unfounded [33]. However, CT angiography and ventilation/ perfusion scans have a relatively low diagnostic yield, detecting pulmonary embolism in less than 20% of cases [34]. Iodine maps generated by dual energy CT have demonstrated high detection rates of segmental perfusion defects [35], but the clinical significance and therapeutic relevance of such microvascular obstructions are not yet known.

**Fig. 7:**
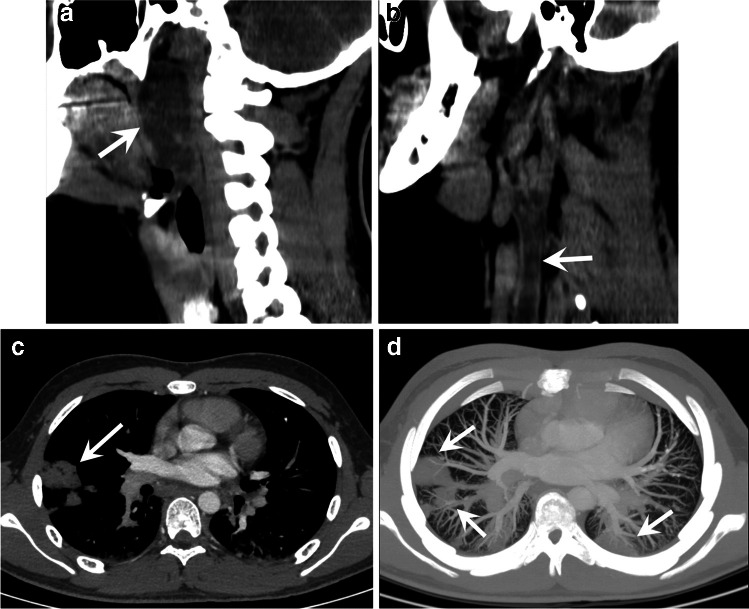
A 16-year-old boy with a peritonsillar abscess, trismus, and hypoxemia. A cervicothoracic contrast-enhanced computed tomography (CT) scan was performed. Sagittal multiplanar reconstruction reformatted images show peritonsillar abscess (*arrow in* **a**) and internal jugular vein thrombophlebitis (*arrow in* **b**). Axial CT image (**c**) and axial lung window maximum intensity projection (MIP) reformatted image (**d**) show multiple bilateral nodules (*arrows*) consistent with septic emboli, as in Lemierre’s syndrome

Other types of embolism include septic and neoplastic thromboembolic disease. In patients diagnosed with a septic condition, pulmonary embolism is a known complication. Septic pulmonary embolism can be a complication of osteomyelitis and deep vein thrombosis [36, 37]. Ear, nose, and throat infections such as Lemierre syndrome [38] can also be the origin of septic embolism (Fig. 7). Pulmonary tumor embolism should be considered in a patient with known neoplasms and clinical suspicion of pulmonary embolism. Oncology patients are at high risk for bland pulmonary embolism due to several factors, including central venous lines, endothelial lesions, and coagulation disturbances associated with chemotherapy, immobilization, or parenteral nutrition. The most common neoplasm associated with tumor pulmonary embolism is Wilms tumor, but it has also been reported in a variety of neoplasms such as osteosarcoma, adrenal carcinoma, or chondrosarcoma. Other less frequent types of pulmonary embolism in children include posttraumatic fat emboli or pulmonary cytolytic thrombi after hematopoietic stem cell transplantation.

### Stenoses, aneurysms and pseudoaneurysms of the pulmonary vessels

Obstruction of the pulmonary vessels may be an emergency in neonates with congenital heart disease as discussed in the previous section. Hereditary disorders such as Alagille syndrome (an autosomal dominant disorder with cholestasis, skeletal anomalies, facial phenotype, and cardiovascular anomalies) or Williams-Beuren syndrome (a partial deletion of chromosome 7q11.23 with characteristic facial features, mental retardation, and cardiovascular anomalies) are frequently associated with pulmonary artery stenosis. Other causes of intrinsic pulmonary vascular occlusion, although very rare, are pulmonary vasculitis such as isolated pulmonary arteritis [39], Takayasu arteritis [40], or Behcet disease. Hughes-Stovin syndrome [41], a rare systemic vasculitis with pulmonary aneurysms, may present as pulmonary artery aneurysms or severe pulmonary hemorrhage.

**Fig. 8:**
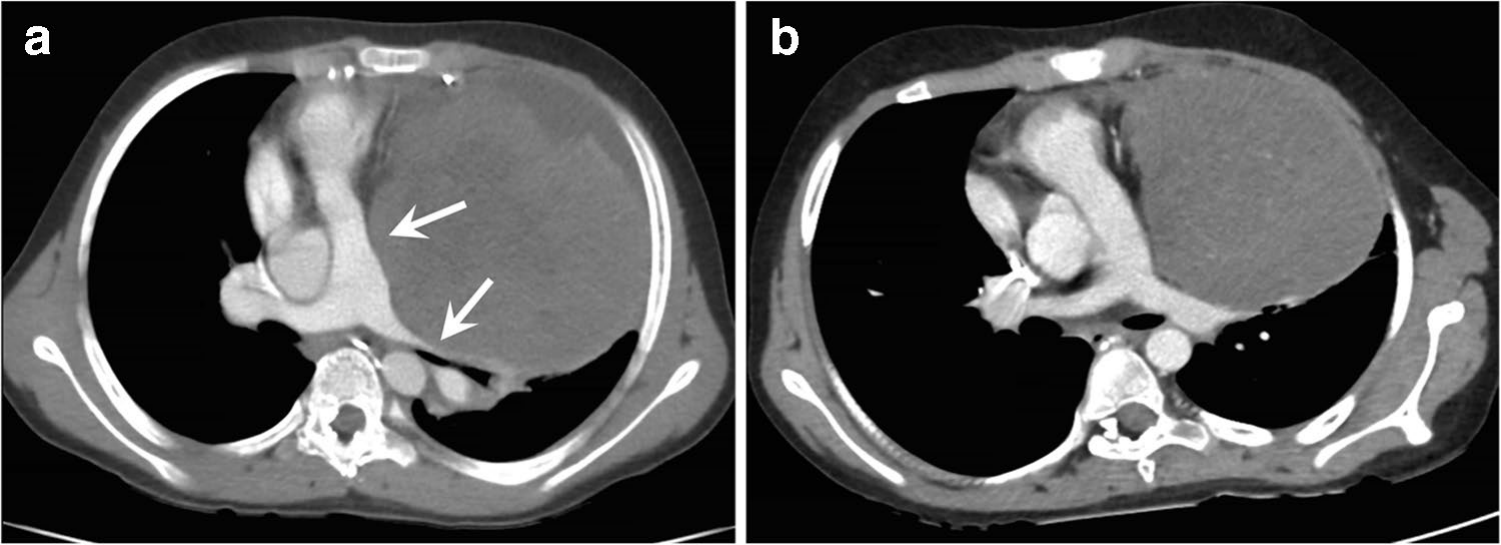
A 13-year-old boy with an anterior mediastinal mass (non-Hodgkin B lymphoma). The patient presented to the emergency department with malaise and dyspnea. A large anterior mediastinal mass was seen on axial contrast-enhanced chest computed tomography (CT) (**a**). Compression with severe stenosis of the left pulmonary artery was seen (*arrows*). **b** Follow-up axial contrast-enhanced CT after chemotherapy 4 weeks later shows significant volume reduction of the mass and resolution of the pulmonary artery compression

Extrinsic compression of the pulmonary arteries and veins includes intrathoracic collections (acute hematomas, post-surgical seromas, abscesses), neoplasms (mediastinal lymphoma, sarcoma or teratoma, chest wall tumors), duplication cysts, or even malformations such as pectus excavatum. Chest pain and dyspnea are the most common clinical manifestations. Tumor encasement or compression can occasionally be treated with stenting, but expectant management with oncologic treatment is generally the main option (Fig. 8).

Pulmonary artery aneurysms and pseudoaneurysms are rare. Iatrogenic lesions are discussed in later sections of this paper. Pulmonary stenoses and aneurysms may be associated with vasculitis such as Takayasu’s or Behcet’s disease. Idiopathic pulmonary aneurysms are extremely rare, but pulmonary artery pseudoaneurysms have been reported in pulmonary infections, especially with aggressive necrotizing organisms such as mucormycosis [42, 43]. Neoplastic pseudoaneurysms have also been described.

### Complications following surgical or interventional treatment

Post-treatment complications are a source of cardiovascular emergencies in children. Some cases are acute life-threatening events and there is no time for CT or MRI. In stable patients, CT is the preferred imaging modality after echocardiography. Complications include aneurysms and pseudoaneurysms, stenoses, bleeding, and infection.

Infections of prosthetic heart valves and cardiovascular devices are also a common cause of pulmonary embolism. Difficulty in assessing conduits and valves with ultrasound or CT angiography makes positron emission tomography-CT (PET-CT) a useful complementary imaging modality [44, 45], not only for the diagnosis of surgical or interventional device infection but also for the detection of pulmonary septic emboli. Post-procedural pulmonary artery pseudoaneurysms and ruptures are rare but potentially fatal complications after stenting and balloon dilatation. When suspected, pulmonary CT angiography is the noninvasive imaging modality of choice. Contrast extravasation, hematoma, and pseudoaneurysm formation are easily detected with CT. The diagnosis of catheter-induced pulmonary artery dissection is usually suspected by echocardiography and confirmed by CT angiography (Fig. 5).

## Imaging of acute acquired cardiovascular diseases

The following sections discuss imaging for some of the more common selected acquired cardiovascular diseases, including trauma, coronary artery abnormalities, myocarditis/cardiomyopathy, and systemic vasculopathy and vasculitides.

### Trauma

Cardiovascular injuries are one of the most common causes of death secondary to trauma, only second to injuries to the central nervous system [46, 47]. Prompt identification and management of critical injuries is paramount to improve survival rate [47]. Cardiovascular trauma can be classified into two major categories: penetrating and blunt thoracic injury. Pediatric cardiac trauma is most commonly due to blunt mechanisms, and these are more frequently secondary to motor vehicle accidents and falls [46, 48–51]. We will focus our discussion on blunt thoracic injury.

Pediatric traumatic chest injuries differ as compared to adults. Children have increased thoracic cage flexibility and compressibility allowing higher deformity of the chest cage. Also, children have increased mobility of the cardiomediastinal structures [50]. These features lead to an increased risk for pulmonary contusions, but less commonly fractures, and, even rarer, aortic injury. Nonetheless, there is approximately 27–40% mortality rate after cardiac injury in children [50].

The mechanisms of injury to the cardiovascular structures due to blunt thoracic injury include direct impact to the precordium with compression of the heart between the sternum and the spine, blast injury, injury due to fractures, or tension pneumothorax. Other mechanisms are due to indirect forces secondary to abdominal trauma, with shearing or displacement of the cardiovascular structures, force injuries transmitted from lower extremity injuries, or due to cardiovolemic change with increased vascular pressure transmitted to the heart [46, 47].

**Fig. 9:**
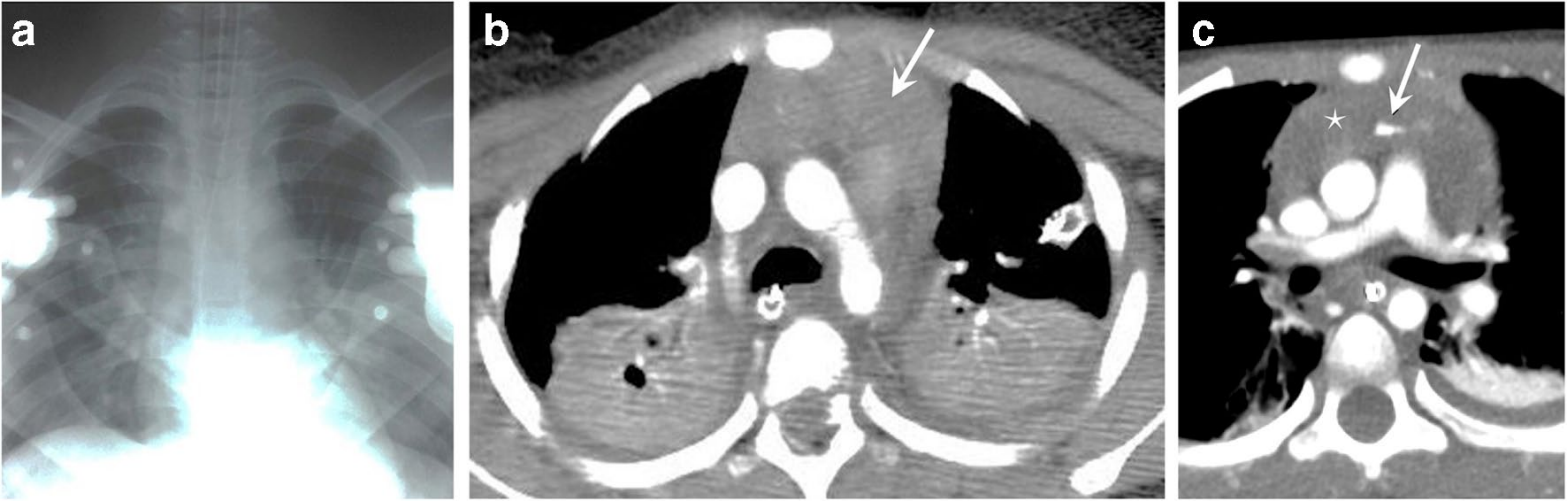
Traumatic non-cardiovascular findings. **a** Anteroposterior chest radiograph in a 9-year-old boy obtained in the trauma bay demonstrates a mild widening of the upper mediastinum with hazy ill-defined increased paramediastinal opacification. The patient is intubated with a feeding tube in place. External devices are obscuring details. **b** A 12-year-old girl where an axial contrast-enhanced computed tomography (CT) shows a large mediastinal hematoma (*arrow*) and consolidations/atelectasis in the dependent aspects of both lungs. **c** A 10-year-old boy with a contrast-enhanced CT showing a large mediastinal hematoma (*asterisk*) with active bleed (*arrow*). Cases courtesy of Dr. David Manson from The Hospital for Sick Children, Toronto, Canada

Patients with chest trauma may present with an abnormal ECG and/or abnormal cardiac enzymes [46, 47]. Findings on radiographs can include pneumopericardium, pericardial effusion with enlargement of the cardiac contour, hydrothorax, hemothorax, pneumothorax, and mediastinal widening, suggesting a hematoma or vascular injury. Echocardiography can demonstrate pericardial effusion, signs of cardiac tamponade, valve dysfunction, or wall motion abnormalities [46, 47]. Contrast-enhanced CT is the gold standard and first-line imaging modality in traumatic cardiovascular injury. Spectrum of CT imaging findings include pneumopericardium, pericardial effusion or hemopericardium, pericardial rupture, myocardial contusion, myocardial tears, cardiac tamponade, cardiac herniation, valve injuries, vascular injuries, or signs of pressure change with right heart strain [52]. Other findings include mediastinal hematomas and other non-cardiovascular findings such as rib or sternal fractures, lung and pleural space findings (Fig. 9), or abdominal or lower extremities injures, translating into cardiovascular injuries [47].

**Fig. 10:**
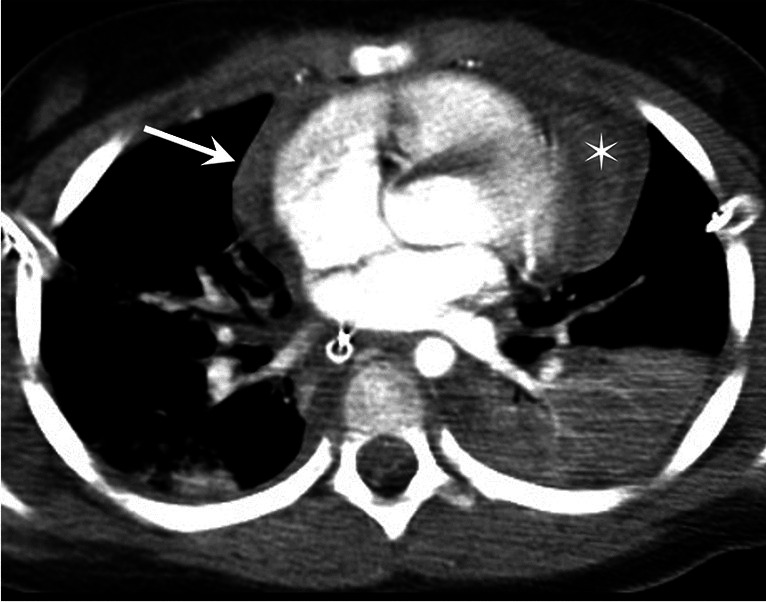
A 12-year-old boy with hemopericardium. Axial contrast-enhanced computed tomography of the thorax demonstrates high density pericardial fluid (*asterisk*) consistent with hemopericardium. Note also a small mediastinal hematoma (*arrow*) and consolidation/atelectasis of the posterior lungs, more confluent on the left. Courtesy of Dr. David Manson from The Hospital for Sick Children, Toronto, Canada

*Pericardial effusion* may occur after acute traumatic injury and in this setting, any pericardial effusion should be considered hemopericardium until proven otherwise [52]. Hemopericardium is usually associated with cardiac chamber rupture and has a high mortality rate, but also can be due to aortic root injury, a myocardial contusion or coronary artery dissection (Fig. 10) [46]. *Pericardial rupture* is rare, seen in about 0.3–0.5% after traumatic injury, and is usually secondary to fractures or deceleration forces [52]. CT can show discontinuity of the pericardium with focal dimpling, pneumopericardium, an empty pericardial sac, cardiac contour or cardiac chamber constriction or deformity, or cardiac luxation [46].

*Cardiac tamponade* results from accumulation of pericardial fluid or hemopericardium, compressing the heart and leading to a decrease in cardiac output, or due to a mediastinal hematoma. Imaging findings include hemopericardium, jugular vein congestion, dilatation of the inferior vena cava and renal veins, and periportal low attenuation fluid [46]. There may be late onset of cardiac tamponade with minor blunt chest trauma in children [53].

The myocardium can be injured from direct impact of the heart against the osseous structures, or due to shearing forces [47]. *Myocardial concussions* show no anatomic or cellular injury, but echocardiography can show focal wall motion abnormalities. *Myocardial contusions* cause anatomic or tissue injury, and can lead to myocardial infarction and elevated cardiac enzymes. Imaging findings can include pneumopericardium, signs of congestive heart failure with pulmonary edema and lung opacities [46]. Echocardiography shows focal increased myocardial echogenicity and systolic hypokinesia [46]. Associated findings include traumatic valvular injuries and ventricular septal defects. Due to its position in the chest, the right ventricle free wall is more frequently injured [46, 47]. Right ventricular injury can cause contractility impairment, and hypovolemia can cause decreased left ventricular preload output.

*Myocardial tears* are an uncommon cause of immediate death after blunt trauma and are a cause of cardiac tamponade and fatal arrhythmias [47, 54]. CT can show focal discontinuity and active contrast extravasation into the pericardial space [47, 55].

*Posttraumatic left ventricular aneurysm*, although rare, has been described in children, along with other findings, such as *interventricular septal aneurysm* and traumatic VSD [48]. Patients can present with features of heart failure, emboli, arrhythmias, and palpitations [48]. Traumatic VSDs are the most frequent traumatic septal injury [47]. These usually occur within a site of a myocardial contusion, near the cardiac apex, in the muscular portion of the interventricular septum [46]. Traumatic VSDs can be seen early immediately after trauma due to mechanical compression, or late when edema disrupts the muscle perfusion with eventual perforation. Traumatic VSD and ventricular aneurysm have also been described in children after blunt injury due to child abuse [56].

Valvular injury can also occur, and due to the higher pressure of the left cardiac chambers, the mitral and aortic valves are at increased risk of injury with valve cusp avulsion or tear [47, 52].

*Coronary artery injuries* are unusual, seen in approximately 2% after traumatic blunt thoracic injuries. When this occurs, the left anterior descending artery is more frequently injured [47, 57].

**Fig. 11:**
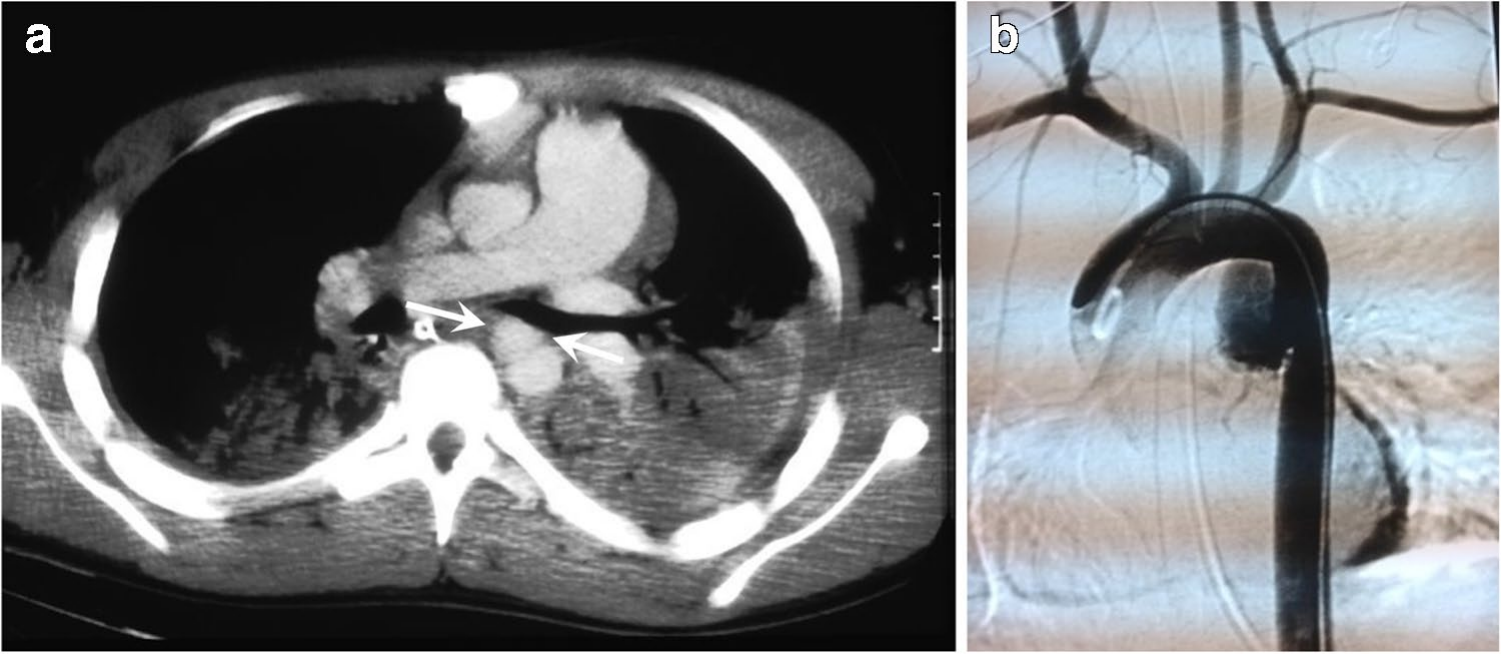
An 8-year-old boy with aortic pseudoaneurysm. **a** Contrast-enhanced computed tomography of the chest demonstrates a pseudoaneurysm in the proximal descending aorta (*arrows*). Bilateral consolidation/atelectasis is also noted in both lungs posteriorly. **b** Lateral view of digital subtraction aortic angiography demonstrates a traumatic pseudoaneurysm in the proximal descending aorta. Cases courtesy of Dr. David Manson from The Hospital for Sick Children, Toronto, Canada

*Aortic injury* is rarely seen in children due to the increased elasticity of the pediatric arterial structures, but can be seen in about 0.05–7.4%, and approximately 90% of children with aortic injury are older than 10 years of age [51, 58]. These are associated with a high mortality rate of about 85% [58]. Aortic injuries frequently occur at the level of the ligamentum arteriosum and include an intimal tear (type I), an intramural hematoma (type II), an aortic pseudoaneurysm (type III) (Fig. 11), and aortic rupture (type IV) [59]. Other findings can include a periaortic hematoma, intimal flaps, vessel wall irregularity or caliber change, vessel occlusion, or active contrast extravasation [60]. In children, an *aortic laceration* can be seen in less than 0.1% after blunt thoracic injury [50, 61–63]. Traumatic aortic dissection may not be as uncommon in childhood or adolescence, with a study reporting aortic dissection in up to 42% [64]. *Pulmonary artery trunk* injury is very rare [47].

**Fig. 12:**
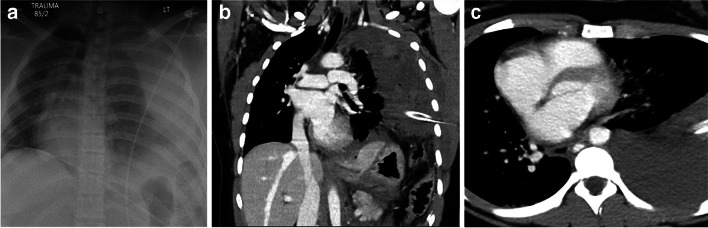
A 16-year-old boy with cardiac displacement due to diaphragmatic rupture. **a** Anteroposterior chest radiograph with electrodes over the thorax. (**b**) Coronal plane of a contrast-enhanced computed tomography (CT) with a chest tube in the left thorax and (**c**) CT in an axial plane. All images demonstrate displacement of the cardiomediastinal structures due to diaphragmatic rupture with herniation of the abdominal contents into the left hemithorax

*Cardiac luxation or herniation* refers to disruption of the cardiac axis or displacement of the heart in the chest (Fig. 12) causing constriction or cardiac torsion. It is the most lethal complication secondary to pericardial rupture with a high mortality rate [46, 47]. Imaging findings include malposition of the heart, bowel herniating into the pericardial sac, or an empty pericardial sac with pneumopericardium [46, 47].

### Coronary artery assessment

Coronary artery assessment is one of the most common indications for urgent cardiac imaging. ECG-gated cardiac CT will almost always be the modality of choice in these situations. Some typical indications include infants with suspected anomalous left/right coronary artery from the pulmonary artery, children post cardiac arrest due to a suspected cardiac cause such as anomalous aortic origin of a coronary artery, patients with an underlying vasculopathy or vasculitis (particularly in Williams syndrome) presenting with new findings or symptoms, or in postoperative patients after coronary reimplantation with persistent ventricular dysfunction or turbulent coronary origin flow by Doppler (e.g., after arterial switch or Ross operations). Occasionally, tubes and devices can also compress or impinge on a coronary artery in postoperative patients with persisting ventricular dysfunction. A few pearls regarding specific scenarios follow.

**Fig. 13:**
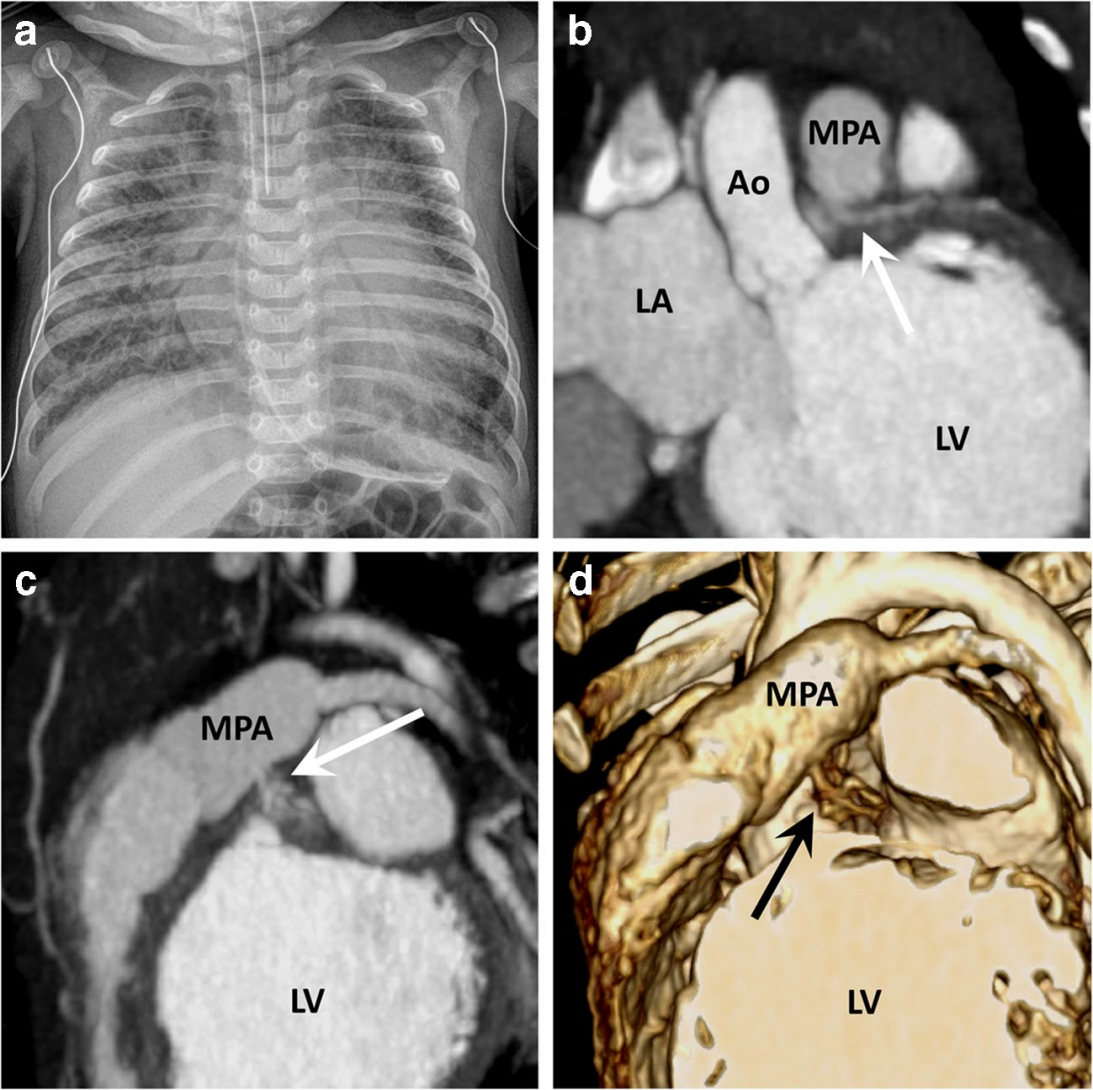
A 9-week-old boy with anomalous origin of the left coronary artery from the pulmonary artery who presented in cardiogenic shock. **a** Anteroposterior chest radiograph shows moderate to severe cardiomegaly with pulmonary edema. An endotracheal tube is near the carina and a nasogastric tube is in the stomach. **b-d** Coronal (**b**) and sagittal (**c**) contrast-enhanced cardiac computed tomography maximum intensity projection and volume rendering technique reconstructions (**d**), show the left main coronary artery (LMCA) arising from the undersurface of the main pulmonary artery (MPA) (*arrows*). This patient also has a fine network of collaterals surrounding the LMCA. Note: the dilated left ventricle (LV) due to ischemic cardiomyopathy. *Ao* aorta, *LA* left atrium

Anomalous left coronary artery from the pulmonary artery is a classic differential consideration in an infant with ventricular dysfunction and heart failure, showing cardiomegaly and pulmonary venous congestion/edema on chest radiographs. The anomalous left coronary artery often arises from the undersurface of the main pulmonary artery (Fig. 13). Because of the lower pressure pulmonary circulation, there is “retrograde” flow from the left coronary artery into the main pulmonary artery, resulting in a left-to-right steal phenomenon and subsequent ischemia. Intrinsic left coronary artery ostial stenosis and right coronary artery, systemic, and bronchial collateralization can mitigate these effects to a degree [65]. The anatomy can often be diagnosed by echocardiography, but in select patients, feed-and-sleep cardiac CT may be required for confirmation of diagnosis.

**Fig. 14:**
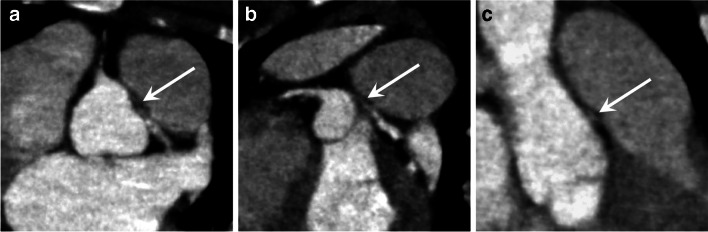
A 12 -year-old boy with an anomalous origin of the left coronary artery from the right aortic sinus who suffered a cardiac arrest while playing basketball. **a**, **b** Multiplanar oblique contrast-enhanced cardiac computed tomography (CT) reconstructions show the anomalous origin of the left main coronary artery from the right aortic sinus arising at an acute angulation with an interarterial course. There is a very thin poorly opacified intramural segment (*arrows*) that crosses across the right-left aortic commissure. **c** Coronal cardiac CT reconstruction shows the interarterial portion in cross section (*arrow*), which is slit-like with loss of pericoronary fat, typical signs of an intramural segment

One of the considerations after new cardiac arrest in older children and teenagers is anomalous aortic origin of a coronary artery. Sudden cardiac events in anomalous aortic origin of a coronary artery typically occur during exertion and more commonly are due to an anomalous left coronary artery from the right aortic sinus. Anomalous right coronary artery from the left aortic sinus is more prevalent overall, but less likely to be symptomatic [66]. A septal coronary course has historically been considered a relatively benign variant, but recently it has been shown that up to 50% of these patients may have inducible myocardial hypoperfusion [67]. Retroaortic and pre-pulmonic coronaries are still considered relatively benign. Some of the more important reporting elements include presence and length of any intramural segment, ostial morphology, and relationship to the aortic valve commissure and intercoronary pillar (Fig. 14).

Concerns for coronary stenosis in infants with vasculopathy or in the postoperative setting after coronary reimplantation may necessitate an urgent cardiac CT for further evaluation. Even in small infants with high heart rates where the spatial and temporal resolution is limited, it is often worthwhile to attempt to visualize the coronaries before deciding if the more invasive gold-standard test of cardiac catheterization requiring general anesthesia should be pursued. This is particularly true in Williams syndrome due to the high risk of sudden death with sedation in these patients [68]. Williams syndrome is caused by an elastin gene mutation that causes arterial media wall thickening and stiffening due to smooth muscle hypertrophy and results in multifocal arterial stenoses. This classically manifests as a characteristic supravalvular aortic stenosis seen in up to 70% of patients, along with peripheral branch pulmonary artery stenoses that can affect 40–75% of patients. The coronaries can be involved in association with the supravalvular stenosis, separately via intrinsic focal ostial stenosis, or indirectly via aortic valve leaflet degeneration and supravalvular tethering in a phenomenon known as “coronary hooding” [69].

Coronary ostial stenosis is also a risk whenever the coronaries are reimplanted, such as after an arterial switch operation or Ross procedure. Urgent cardiac CT may be required in the immediate postoperative period when there is persistent ventricular dysfunction or coronary concerns by echocardiography. Visualization of the coronary origins by cardiac CT is often useful to show or exclude significant proximal coronary stenosis, kinking, or stretching. Coronary assessment by cardiac CT can be particularly successful after arterial switch operation, as in these patients the coronaries are typically reimplanted higher in the neoaorta, which is a region less susceptible to cardiac motion.

### Myocarditis/cardiomyopathy

The diagnosis of viral myocarditis in the pediatric population can usually be made based on clinical, ECG, serologic, and echocardiographic findings alone. CMR is indicated when the diagnosis is in doubt and for potential prognostication [9] and can be obtained in the semi-urgent acute setting after patients are stable. The most important diagnosis to exclude is acute coronary ischemia; however, congenital cardiomyopathy and other rheumatologic, granulomatous, or neoplastic causes of myocardial inflammation can also rarely mimic typical viral myocarditis. This picture has been even more complicated with the recent rise of myocarditis due to coronavirus disease-19 (COVID-19), COVID-19 vaccine-adjacent myocarditis, and multisystemic inflammatory disease in children (MIS-C) myocarditis (± coronary involvement). Our understanding of COVID-19-related myocardial injury is continuing to evolve and a detailed review is beyond the scope of this article.

**Fig. 15:**
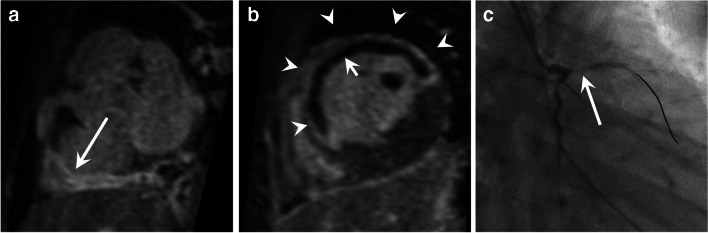
An 11-year-old boy with a myocardial infarction who presented in hypertensive crisis with cardiac dysfunction, initially suspected to be an infectious or inflammatory myocarditis. **a** Short-axis magnetic resonance 3-dimensional high-resolution late gadolinium enhancement reconstructions show irregular wall thickening and enhancement of the right coronary artery (*arrow*), suggestive of a coronary vasculitis. **b** There is also transmural late gadolinium enhancement in the left anterior descending (LAD) territory (*arrowheads*) with significant central subendocardial microvascular obstruction (*short arrow*), consistent with a myocardial infarction. The LAD itself was not able to be well visualized (not shown). **c** Subsequent right anterior oblique cardiac catheterization projection shows a wire bypassing a proximal LAD occlusion

The modified Lake Louise diagnostic criteria for myocarditis are the presence of (1) myocardial edema via T2-mapping or T2-weighted images and (2) non-ischemic myocardial injury via T1-mapping or late gadolinium enhancement. Supportive criteria include evidence of pericarditis and findings of pericardial effusion or ventricular dysfunction [70]. An ischemic cause must be suspected when the late gadolinium enhancement pattern is subendocardial or transmural and confined to a coronary perfusion territory (Fig. 15). If there is any clinical possibility of either an ischemic cause or MIS-C, coronary magnetic resonance angiography should be included in the protocol. Using a gadolinium-enhanced, cardiac-gated, respiratory-navigated, 3D inversion recovery gradient echo (GRE) sequence for both coronary magnetic resonance angiography and late gadolinium enhancement can be very useful in this scenario [71–73].

### Systemic vasculopathy/ vasculitis

**Fig. 16:**
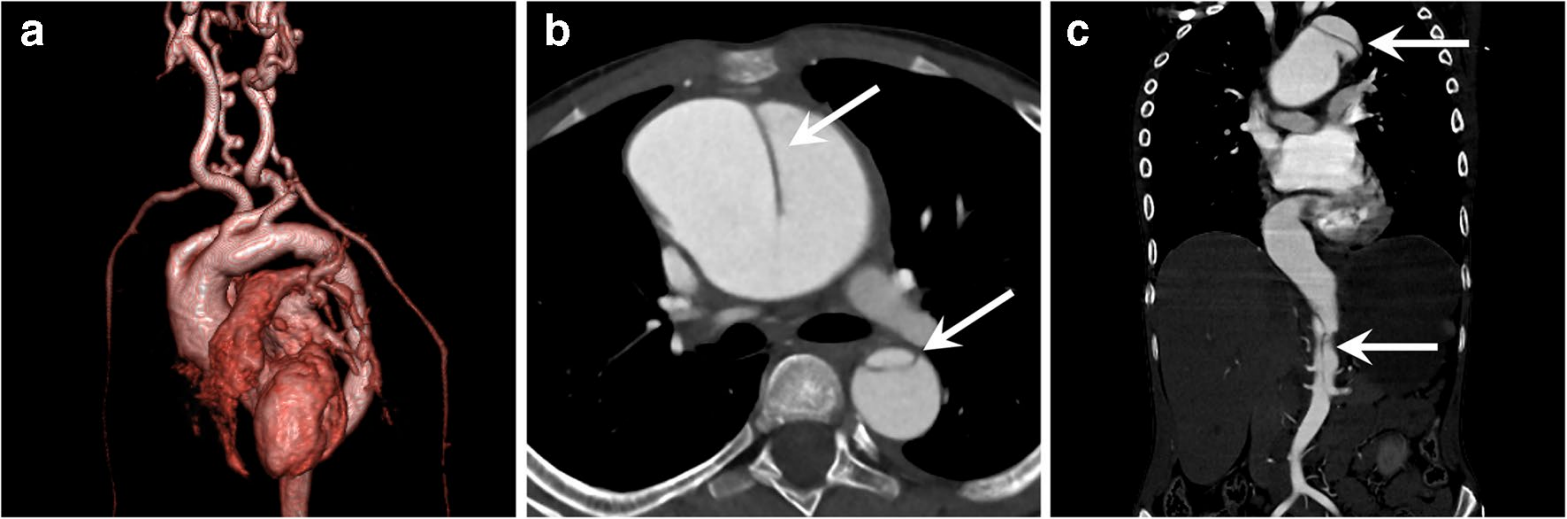
A 4-year-old boy with Loeys-Dietz syndrome complicated by aortic dissection. **a** Contrast-enhanced computed tomography (CT) parasagittal-oblique volume rendering projection shows a dilated and tortuous aorta, along with severe tortuosity of the head and neck vessels, especially of the vertebral arteries. **b, c** Axial (**b**) and coronal (**c**) CT images 4 years later show an aortic dissection flap (*arrows*) extending from the ascending aorta to infrarenal abdominal aorta, consistent with a type-A dissection

Systemic vasculopathies and vasculitides may occasionally require acute imaging for complications of the disease. Of the inherited vasculopathies, the entities of connective tissue disorders most commonly present with acute complications. These include Marfan syndrome, Loeys-Dietz syndrome, and vascular Ehlers-Danlos syndrome. Loeys-Dietz syndrome is an autosomal dominant connective tissue disorder caused by dysregulation of transforming growth factor beta (TGF-β). Vascular features of Loeys-Dietz syndrome include aggressive arterial tortuosity and development of aneurysms (Fig. 16), which can occur beyond the aortic root (unlike Marfan syndrome). Loeys-Dietz syndrome has a higher risk of dissection and rupture, which can occur at smaller sizes. The vertebral tortuosity index, which is a ratio of the vertebral artery length to straight-line cranio-caudal length, is an imaging biomarker for cardiovascular prognosis in connective tissue disorders and is particularly useful for Loeys-Dietz syndrome [74]. Ehlers-Danlos syndrome encompasses a spectrum of genetic disorders with underlying defective collagen synthesis. Vascular Ehlers-Danlos syndrome mainly affects the large and medium systemic arteries. The vessels in vascular Ehlers-Danlos syndrome are extremely friable and very susceptible to the development of pseudoaneurysms and dissections regardless of size, making these children incredibly difficult to manage. Even minor trauma can lead to injury and any intervention can be complicated by additional aneurysms, rupture, or dissection [75].

The most common vasculitides to affect the pediatric population are Takayasu arteritis and Kawasaki disease. Takayasu arteritis is a chronic idiopathic granulomatous large vessel vasculitis that predominantly affects the aorta and major branches. It most commonly results in stenoses, but can also cause complete occlusions, aneurysms, and dissections. Childhood Takayasu has high morbidity with greater involvement of the abdominal aorta than in adults. Hypertension, which is mainly renovascular, is present in 70–80% of pediatric patients, and 10–35% of children have lower limb claudication [76]. Imaging will show vessel wall thickening, edema, and enhancement. Kawasaki disease is a small- and medium-sized vessel vasculitis that presents in early childhood. The most serious complication of Kawasaki disease is a coronary arteritis that can cause coronary aneurysms and lead to thrombus and stenoses. Myocardial infarction can occur even many years after the disease and concern for acute coronary syndrome may require acute cross-sectional imaging [77]. On long-term surveillance, the affected coronary arteries typically have persistent hyperenhancement, which could potentially be related to chronic luminal myofibroblastic proliferation and/or fibrosis.

## Conclusion

Pediatric cardiovascular emergencies encompass many different entities ranging from acquired traumatic and nontraumatic injuries congenital anomalies of the heart and mediastinal vessels. In the acute setting echocardiography, chest radiography and computed tomography angiography are first-line examinations while there are specific indications for cardiac and thoracic MR providing morphological as well as functional data.

## Data Availability

Not applicable to this article as no datasets were generated or analyzed during the current study.
